# Mapk14 is a Prognostic Biomarker and Correlates with the Clinicopathological Features and Immune Infiltration of Colorectal Cancer

**DOI:** 10.3389/fcell.2022.817800

**Published:** 2022-01-24

**Authors:** Dan Wang, Li Peng, Li Hua, Jiaxiang Li, Yifei Liu, Yanhong Zhou

**Affiliations:** Department of Medical School of Facial Features, Hubei University of Science and Technology, Xianning, China

**Keywords:** MAPK14, colorectal cancer, bioinformatics, immunohistochemistry, biomarker

## Abstract

**Background:** Colorectal cancer (CRC) is one of the most common gastrointestinal tumors, ranking in the top 5 of all common tumors in terms of incidence and mortality. However, the mechanisms driving the evolution of colorectal cancer remain unclear. Therefore, we investigated the association between *Mapk14* expression and clinicopathological and tumor-infiltrating immune cells.

**Methods:** In this study, we collected CRC patient data from The Cancer Genome Atlas (TCGA), compared the expression level in CRC and normal colorectal tissue using the Wilcoxon rank sum test and assessed the relationship between *Mapk14* and clinicopathological features using the Welch one-way ANOVA test. Kaplan-Meier and timeROC GSE17537 datasets were obtained from the Gene Expression Omnibus (GEO) dataset to assess the prognostic impact of the *Mapk14* gene on colorectal cancer. Second, we further explored the methylation level of *Mapk14* and its influencing factors. Single-cell sequencing of *Mapk14* in the tumor immune microenvironment (TIME) was analyzed using the GSE108989 dataset. Further analyses based on the TIMER method were performed to assess the correlation between *Mapk14* and tumor immune infiltration, immune checkpoints, tumor mutational load and microsatellite instability. Finally, the results of the bioinformatics analysis were verified by an immunohistochemical analysis.

**Results:** The results showed that the expression of *Mapk14* was upregulated in CRC tumor tissues compared with normal colorectal tissues and the high expression of Mapk14 was associated with poor clinicopathological features and poor prognoses in the CRC array. In addition, cg05798012 and cg25375420 of *Mapk14* are the main DNA methylation sites affecting OS. Single-cell sequencing of the tumor immune microenvironment showed that the abundance and cell state of dysfunctional T cells changed greatly. Importantly, the abnormal overexpression of *Mapk14* in colorectal cancer is related to the level of immune infiltration of immune cells (including CD8^+^ T cells, neutrophils, dendritic cells, B cells, CD4^+^ T cells, and macrophages). The high expression of Mapk14 was significantly correlated with immune checkpoints (including *SIGLEC15*, *TIGIT*, *LAG3*, *CTLA4* and *PDCDILG2*), while the high expression of *Mapk14* was negatively correlated with TMB and MSI but mostly positively correlated with drug sensitivity. Finally, the immunohistochemical results confirmed that the clinical stage (Ⅰ, Ⅱ, Ⅲ and Ⅳ) and M stage (M0 and M1) affected the abnormally high expression of Mapk14.

**Conclusion:** A comprehensive bioinformatics study and experimental validation revealed that Mapk14 could serve as a novel prognostic biomarker associated with immune infiltration and pharmacotherapy and may represent a potential therapeutic target for the treatment of CRC.

## Introduction

Colorectal cancer (CRC) is one of the most common malignant tumors in the world, and recent reports indicated that its incidence rate is second only to that of lung cancer (La Vecchia and Sebastián, 2020; Chen et al., 2021). CRC is a complex disease with a variable clinical course and important treatment response differences, even in tumors with similar histopathological characteristics. The reported recurrence rate was 33% in patients with stage II and III CRC and 73% in patients with metastatic stage IV CRC who underwent potentially curative resection (Pranteda et al., 2020). Although great progress has been made in the diagnosis and treatment of colorectal cancer, the prognosis of patients is still worrying (Wang Y. F. et al., 2021). Therefore, early detection, early diagnosis and early treatment are the keys to improving the prognosis of colorectal cancer patients and tumor markers have wide applicability for judging prognosis, predicting curative effects and monitoring recurrence (Duffy et al., 2014).

Tumorigenesis is related to the characteristics of cancer cells themselves and plays an important role in the immune system*.* Immune cells are important components of the tumor microenvironment and play an immune surveillance role (Alvarez-Errico, 2021). The main immune cells include T lymphocytes, B lymphocytes, macrophages, natural killer cells (NK cells), neutrophils and dendritic cells. Immune checkpoints play a key role in maintaining immune homeostasis. When an inhibitory antibody is targeted to the immune checkpoints of cytotoxic T lymphocyte-associated protein 4 (*CTLA-4*) and programmed death protein 1 (*PD-1*), it can restore immune competence in cancer patients (Wang Y. et al., 2021). Immunosuppressive mechanisms of the tumor microenvironment may represent potential targets for future immunotherapy, especially in the context of the current lack of effective treatments and low survival rates for colorectal cancer. Therefore, the immunophenotype of tumor-immune interactions must be determined and novel biomarkers and therapeutic targets for colorectal cancer must be identified.

P38 mitogen-activated protein kinase (P38*MAPK*) is a class of highly conserved intracellular serine and threonine protein kinases. As an important member of the *MAPK* family, P38*MAPK* has four isoforms, namely, P38α*(Mapk14)*, P38β*(Mapk11)*, P38γ*(Mapk12)* and P38δ*(Mapk13)*, and plays an important role in the regulation of apoptosis. *Mapk14* is usually ubiquitously expressed at high levels in the same tissue, while Mapk11 expression is low, and the expression of *Mapk12* and *Mapk13* is more restricted. Among these isoforms, P38α encoded by the *Mapk14* gene is the key to regulating apoptosis (Igea and Nebreda, 2015). More than 100 proteins can be directly phosphorylated by p38α, and a large proportion of them are involved in the expression and regulation of genes. P38α is probably the most characteristic isoform, and it has a broad gene expression profile, is involved in many cellular physiological activities and pathologies, is highly active in a variety of tumors and thus can act as a tumor promoter or repressor; therefore, it has been the focus of many studies (Cuadrado and Nebreda, 2010; McNamee et al., 2010; Corre et al., 2017; Liu et al., 2020). Studies have shown that *Mapk14* overexpression in breast (Limoge et al., 2017), ovarian (Song et al., 2017), and cancer (Zhao et al., 2019) cells promotes tumorigenesis and progression. However, the biological function of *Mapk14* gene in colorectal cancer has not been elucidated.

**FIGURE 1:**
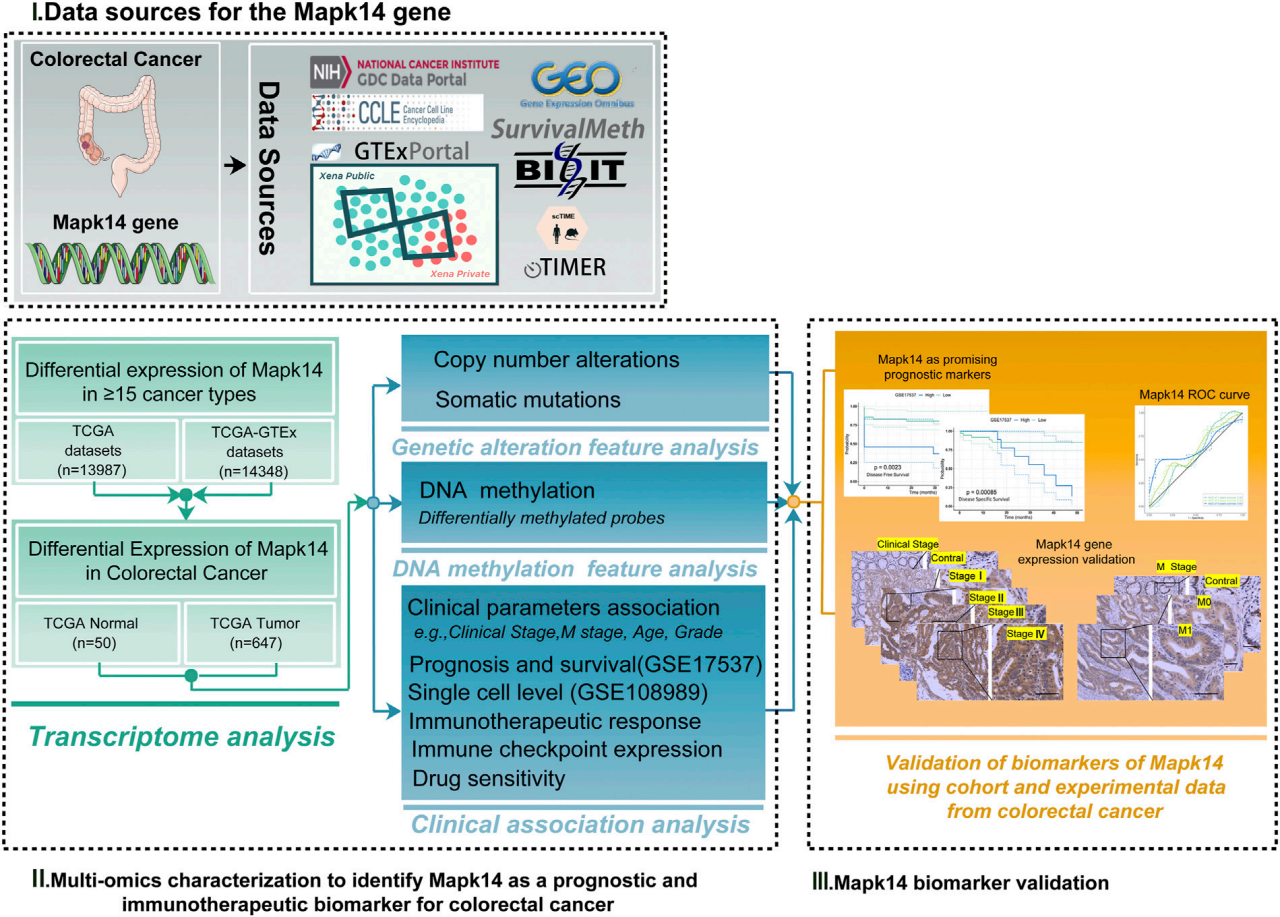
Analytical flowchart of this study. First, we explored the differential expression of Mapk14 in ≥16 tumor types. Then the multi-omic profile of Mapk14 in colorectal cancer was analyzed, and we identified Mapk14 as a prognostic and biomarker for colorectal cancer. Finally, we investigated the functional significance of Mapk14 in CRC and validated the Mapk14 biomarker using IHC experimental data.

Therefore, we determined the association between genetic alterations in *Mapk14* and clinical events (e.g., clinical stage, M-stage, and immunotherapy response) by multi-omic profiling, using large multi-omic data from the same tumor. We comprehensively investigated *Mapk14* expression levels as well as DNA methylation, gene mutations and copy number changes in colorectal cancer. In addition, we investigated the correlation of *Mapk14* with different levels of immune cell infiltration, our research further may provide additional evidence for prognostic biomarkers and therapeutic targets in CRC (Figure 1).

## Materials and Methods

### Mapk14 Genes Differentially Expressed Data

TCGA^1^ (The Cancer Genome Atlas) RNA-seq data (Supplementary Table S1) and mRNA expression data for 16 tumors with paired normal tissue samples were downloaded from the GDC (Genomic Data Commons) data portal (Zhou et al., 2020; Frost et al., 2020; Izzi et al., 2020; Zhang Q. et al., 2020). Gene expression matrices of cell lines from 17 different tumors were obtained from the Cancer Cell Line Encyclopedia (CCLE) dataset^2^ (Ghandi et al., 2019). In addition, we investigated *Mapk14* mRNA expression in cancer and normal tissues using comprehensive datasets from the TCGA and GTEx^3^ (Genotype-Tissue Expression) databases. Visualization was performed by the R package (v3.6.3) “ggplot2” (v3.3.3). Next, raw counts of colorectal cancer RNA sequencing data (level 3) and corresponding clinical information were used for further analysis. A Sankey diagram was built based on the R software package “ggalluval” (Zeng et al., 2019).

### Mapk14 Genes and Survival Analysis

To investigate the prognostic impact of *Mapk14* mRNA on colorectal cancer samples, we downloaded the GSE17537 dataset from the GEO^4^ data portal (https://www.ncbi.nlm.nih.gov/geo/) (Smith et al., 2010). Using R software, we screened the data for *Mapk14* gene expression, survival time and survival status; Kaplan-Meier curves were visualized using the “survival and survminer” package; and an R package “timeROC” analysis was performed to compare the prognostic accuracy and risk score of *Mapk14* mRNA (Supplementary Table S2) (Lin et al., 2020; Zhang Z. et al., 2020).

### Mapk14 Gene Mutations and Methylation Analysis

To investigate the main factors affecting the aberrant overexpression of *Mapk14* in CRC, the relationships among *Mapk14* mRNA expression, methylation levels, copy number and mutations in TCGA-COAD-READ samples was analyzed using the UCSC Xena database^5^(Supplementary Table S3) (Goldman et al., 2019). Before performing these analyses, we set the following conditions: the sample type included solid normal tissue and primary tumor, *Mapk14* was indicated using “RNAseq-IlluminaHiSeq”, DNA methylation was indicated using “Methylation450K”, copy number was indicated using “(gene-level)-gistic2”, and somatic mutations were indicated using “(SNP and INDEL)-MC3 public version”. In addition, the prognostic value of *Mapk14* methylation levels in CRC was analyzed using the SurvivalMeth^6^ (Zhang et al., 2021) and MethSurv^7^ (Modhukur et al., 2018) databases (Supplementary Table S4).

### Mapk14 and Tumor Microenvironment Analysis of Colorectal Cancer

The scTIME^8^ web portal is a database (Supplementary Tables S5–S6) (Hong et al., 2021) for exploring and analyzing TIMEs with time-specific analysis modules at the single-cell level. We used the “NormalizeData” function with the “LogNormalize” method and the “10,000” scale factor to normalize the GSE108989 CRC t cell dataset and applied the ‘RunUMAP’ function by setting dims to “1:20” to perform nonlinear dimension reduction on the data. Then, the proportions and correlations of different cell types in colorectal cancer patients were calculated. In addition, in the LR interactive network module, we use CellPhoneDB v2 to predict the communication between cell types and set the parameter as “-iterations = 1000-threads = 5-counts-data gene_name-subsampling-subsampling-logfalse-subsampling-num-cells1000-threshold 0.01”.

### Correlation Analysis of Mapk14 Expression With Immune Infiltration, Tumor Mutational Load and Microsatellite Instability

For a reliable immune assessment, we used the TIMER algorithm to analyze immune scores and visualized them with the R “ggplot2” and “pheatmap” packages (Newman et al., 2015; Becht et al., 2016; Li et al., 2016; Aran et al., 2017; Racle et al., 2017; Finotello et al., 2019; Sturm et al., 2019; Hu et al., 2020; Li et al., 2020; Liu et al., 2021). Next, we analyzed the infiltration of six immune cells using the TIMER^9^ (tumor immune estimation resource) online tool. In addition, we also analyzed immune checkpoint-related genes by the Wilcoxon test (Supplementary Tables S7–S8) (Ravi et al., 2018; Wang et al., 2019; Zeng et al., 2019; Yi et al., 2020). Spearman’s correlation analyses of tumor mutational burden (TMB) and microsatellite instability (MSI) (Supplementary Table S9) analysis results were performed to describe the correlation between quantitative variables without a normal distribution. A *p* value < 0.05 was considered statistically significant (Thorsson et al., 2018).

### Immunohistochemical Staining of Mapk14 (IHC)

In this study, 35 CRC tissue microarrays from 14 males and 21 females with different clinical stages and M-stages (including cancerous and paraneoplastic tissues) were selected (Supplementary Figure S6). These tissue microarrays (CRC-1402)were purchased from Wuhan Aiwei Biotechnology Corporation, China, and they had a core diameter of 1.5 mm, thickness of 4 μm, and 7 × 10 double core array. Here, we used *Mapk14* (P38 *MAPK*) markers to analyze the expression based on the clinical stage and M-stage of colorectal cancer. Following the standard immunohistochemistry kit procedure for the Dako REAL EnVision detection system (Dako, in review, USA), the sections were soaked in a 30% hydrogen peroxide-methanol mixture for 10 min, placed in a staining tank with sodium citrate, boiled for 10 min, and naturally recovered to room temperature. Tissue microarrays were immunostained with an antibody against P38 *MAPK* (1:200, Proteintech, 14064-1-AP). After incubation overnight at 4°C and washing, the tissues were incubated with a 1:200 dilution of HRP-conjugated secondary antibody (Santa Cruz Biotechnology, Santa Cruz, CA, United States). The IHC signal was visualized by DAB substrate development and counterstained by hematoxylin. Each section was randomly sampled at ×200 magnification. This study was approved by the Ethics Committee of Hubei University of Science and Technology, Xianning, Hubei, China.

### Statistical Analysis

All gene expression data were normalized by log2 transformation. Normal and cancerous tissues were compared based on the Wilcoxon rank sum test; and the significance of *Mapk14* expression in patients with different clinical stages and M stages was assessed by the Welch one-way ANOVA test, with *p* < 0.05 considered statistically significant. Patient survival was analyzed using Kaplan-Meier curves and timeROC curves according to *Mapk14* expression levels, and correlation analyses between the two variables were performed using the Spearman test, with *p* < 0.05 considered significantly different. All statistical analyses were processed by R software (version 3.6.3).

SPSS 26.0 software was used for the IHC data analysis. The χ^2^ test was used to analyze the *Mapk14* expression differences in CRC tumor tissues and adjacent tissues (*p* < 0.05 was considered statistically significant).

## Results

### Abnormally High mRNA Expression of Mapk14 in Human Cancer

**FIGURE 2:**
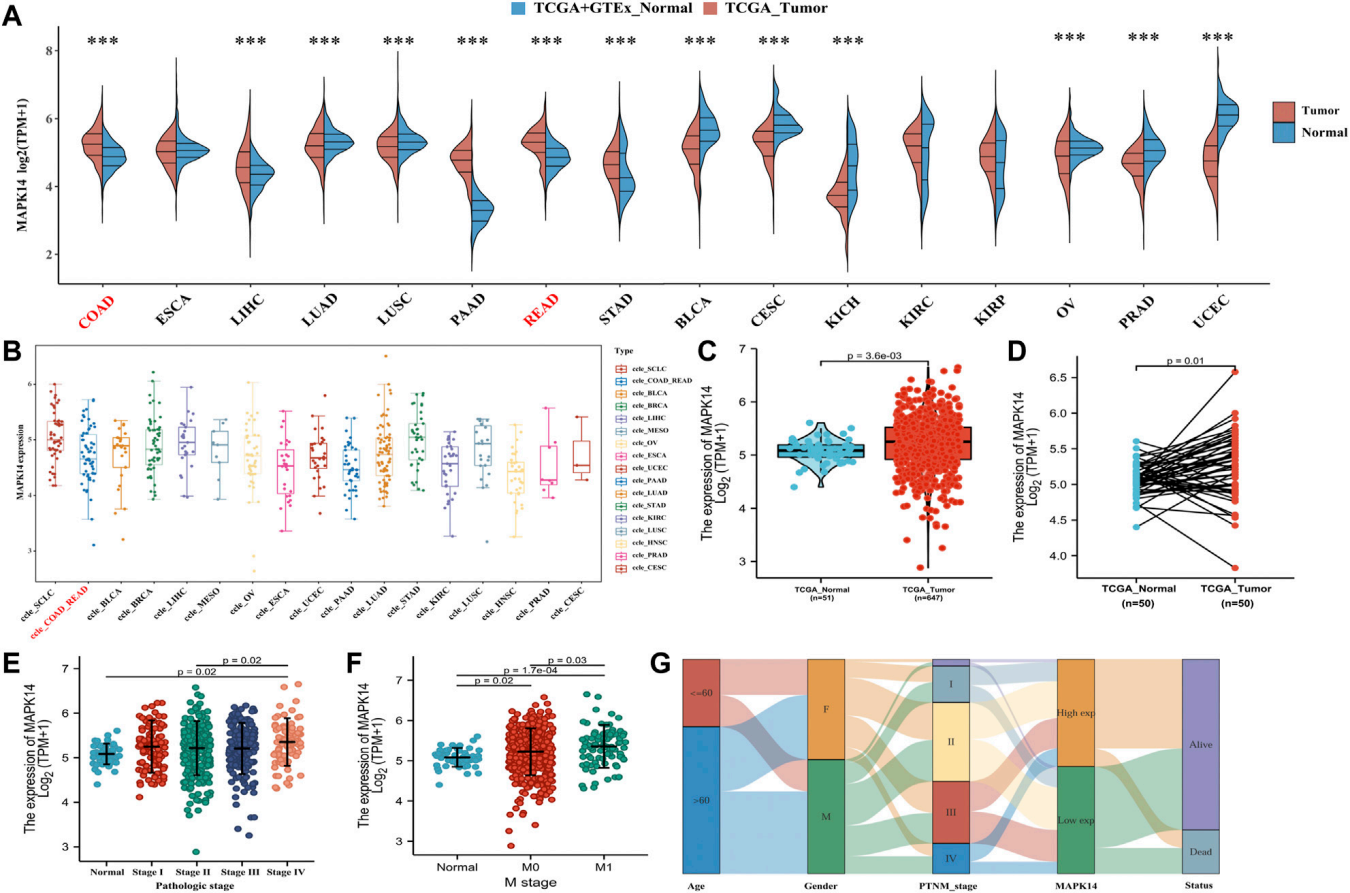
Expression analysis of Mapk14 in multiple cancers and the relationship between clinical and pathological features of Mapk14. **(A)** Expression distribution of the Mapk14 gene in 16 human cancers was investigated based on TCGA cancer data and TCGA and GTEx normal data. The horizontal axis represents different tumor tissues, the vertical axis represents the expression distribution of this gene, and different colors represent different groups. The significance of the two groups of samples passed the Wilcoxon test. **(B)** Expression distribution of the Mapk14 gene in different tumor tissues. The horizontal axis represents different groups of samples, and the vertical axis represents the Mapk14 gene expression distribution. **(C–D)** Comparison of Mapk14 expression between TCGA-CRC data and corresponding TCGA normal data. **(E)** Mapk14 expression in different pathological stages (stages I, II, III and IV) was analyzed based on the TCGA database. **(F)** Expression of Mapk14 in patients with different M-stages was analyzed based on TCGA data. **(G)** Mapk14 expression was associated with different clinical features according to Sankey plots. *p < 0.05, **p < 0.01, and ***p < 0.001.

To investigate the possible role of *Mapk14* in carcinogenesis, we first analyzed its expression in 16 human cancers. *Mapk14* was significantly upregulated in five cancer types (namely, COAD, LIHC, PAAD, READ and STAD) and significantly downregulated in eight cancer types (namely, LUAD, LUSC, BLCA, CESC, KICH, OV, PRAD and UCEC). However, significant differences were not observed for *Mapk14* in ESCA, KIRC and KIRP (Figure 2A). In addition, we downloaded tumor cell line data from the CCLE database to analyze *Mapk14* expression. The results showed that the expression of ccle_COAD-READ remained at 4-6, which was higher than that of ccle_BLCA, ccle_MESO, ccle_ESCA, ccle_UCEC, ccle_PAAD, ccle_KIRC, ccle_LUSC, ccle_PRAD and ccle_CESC (Figure 2B). Second, we verified the expression of *Mapk14* in 51 paracancerous samples and 647 CRC samples in the TCGA-COAD-READ dataset by the Wilcoxon rank sum test method. The results showed that *Mapk14* expression was significantly higher in the unpaired CRC samples (*p* = 0.0036) (Figure 2C) and significantly differed in the paired samples (*p* = 0.01) (Figure 2D), and this finding was consistent with the above results. In summary, *Mapk14* may play a key regulatory role in the carcinogenesis of colorectal cancer.

Next, we further evaluated the relevance of *Mapk14* in the clinicopathological features of colorectal cancer patients based on the clinical pathologic stage and M stage. *Mapk14* expression was higher in stage I, stage II, stage III and stage IV (*p* = 0.02) than in normal tissues (Figure 2E) and significantly higher in the M0 (*p* = 0.02) and M1 (*p* = 0.00017) stages than in normal tissues (Figure 2F). A Sankey diagram was used to show the trend of high and low expression of the *Mapk14* gene for different clinical characteristics, such as stage, age and sex, in colorectal cancer patient samples and the survival of colorectal cancer patients (Figure 2G). These results suggest that *Mapk14* is highly expressed in colorectal cancer tissues and can predict the malignancy of colorectal cancer patients.

### Prognostic Value of Mapk14 in Colorectal Cancer

**FIGURE 3:**
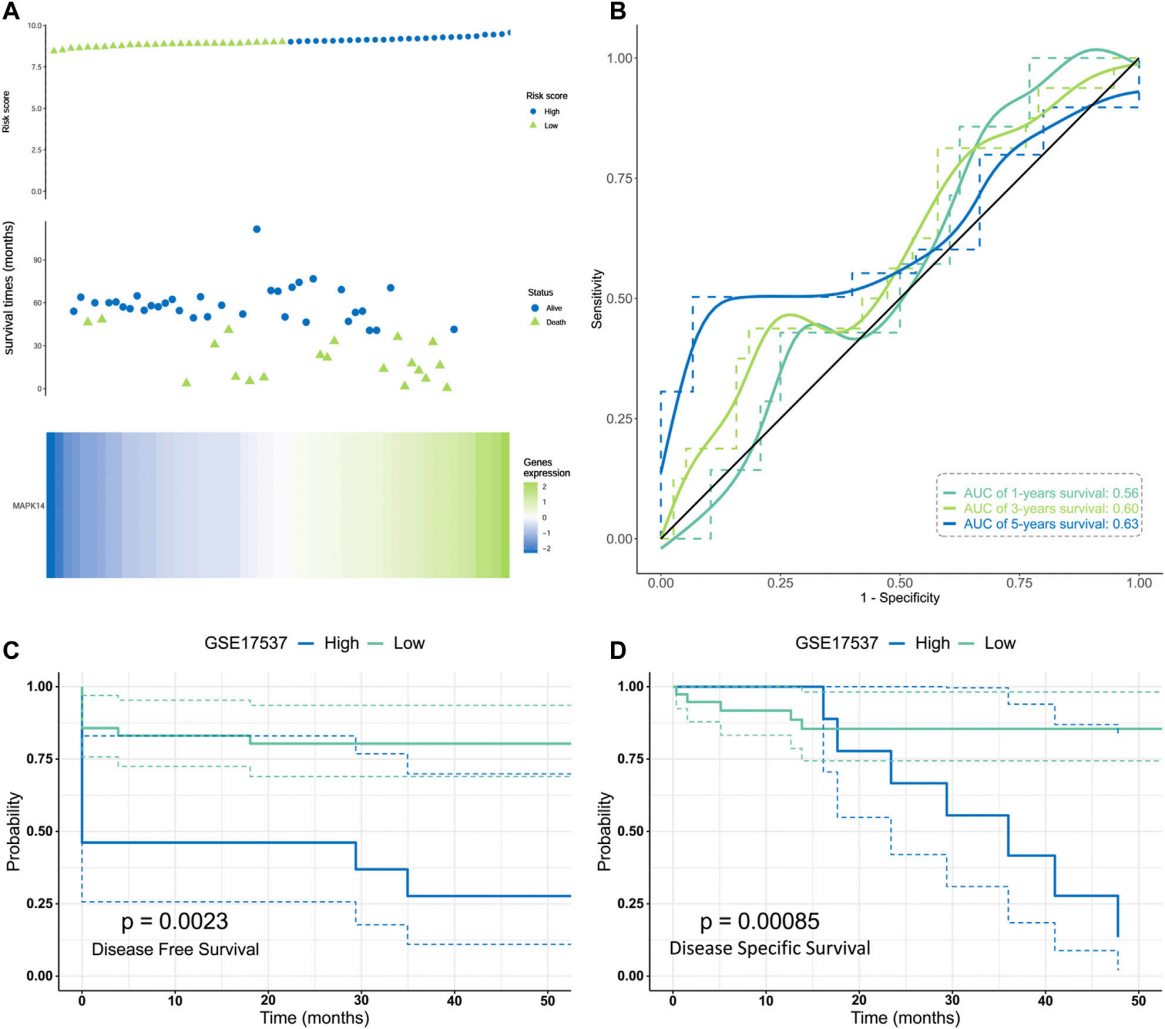
Validation of the risk model in the GES17537 cohort.**(A)**Distribution, survival time and survival status of CRC patients and Mapk14 gene expression in the GSE17537 cohort based on median risk score. **(B)** ROC curve and AUC of Mapk14 gene. **(C–D)** Kaplan-Meier curves for the DFS/DSS of patients in the high- and low-risk groups. .

To investigate whether *Mapk14* expression can be used as a prognostic marker for colorectal cancer patients, we evaluated the distribution of *Mapk14* expression levels and its relationship with patient Disease-free survival (DFS) and Disease Specific Survival (DSS) using the GSE17537 dataset. According to the prognostic impact of *Mapk14* on colorectal cancer samples, the samples were ranked from high to low *Mapk14* expression and the cancer samples were divided into high and low expression groups. A combined risk curve and survival status analysis revealed that the corresponding middle scatter plot from left to right presented increasing death and shorter survival time trends in CRC patients, which indicated that the mortality rate in the high *Mapk14* expression group was higher than that in the low expression group (Figure 3A). However, the Kaplan-Meier survival analysis showed that high *Mapk14* expression was significantly associated with DFS(*p* = 0.0023) and DSS(*p* = 0.00085) prognosis in CRC patients (Figures 3C,D). To observe the predictive value of *Mapk14* mRNA levels on prognosis, we assessed *Mapk14* mRNA expression by a ROC curve model. The area under the curve (AUC) was more accurate when it was between 0.5 and 1, and it was assessed to predict the 1-,3-, and 5-years risk of CRC patients (1 year, AUC = 0.56; 3 years, AUC = 0.60; 5 years, AUC = 0.63) (Figure 3B). In conclusion, the *Mapk14* prognostic model has some predictive potential for CRC patients.

### Factors Affecting Mapk14 Gene Expression in CRC

**FIGURE 4:**
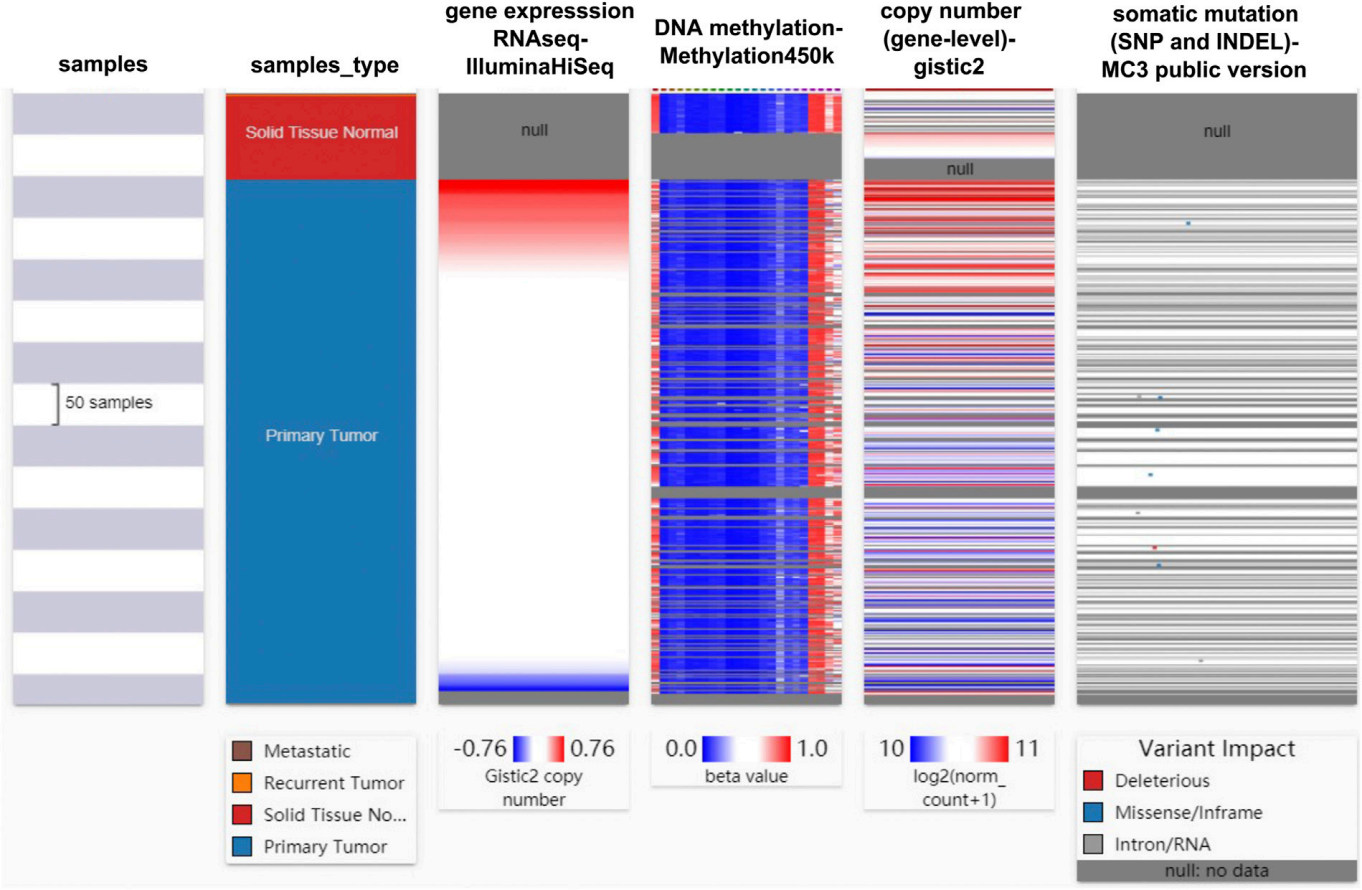
Heat map of Mapk14 mRNA expression, methylation, copy number and somatic mutations in CRC patients and normal tissues. Data are from the TCGA-COAD-READ and include 736 samples in total.

**FIGURE 5:**
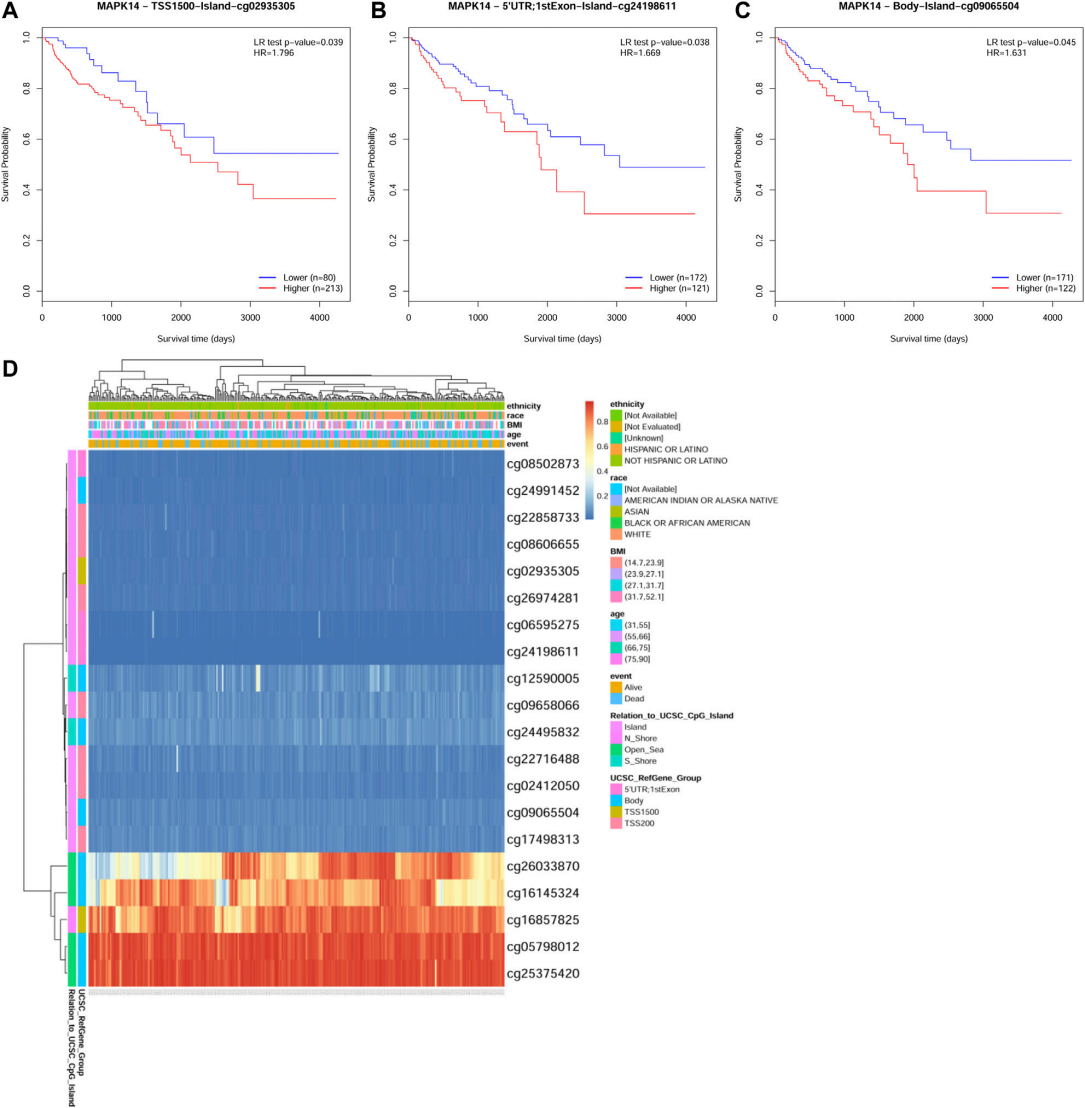
MethSurv database analysis of the effect of MAKK14 gene methylation levels on the prognosis of COAD. **(A–C)** Kaplan-Meier survival of Mapk14 promoter methylation in COAD was observed (cg02935305, cg24198611 and cg09065504. *p* < 0.05). **(D)** Visualization of the relationship between methylation levels and Mapk14 expression in COAD.

To identify the main factor influencing *Mapk14* gene expression, we used online tools for mutation, methylation and CNV alteration analyses. We used three methylation analysis tools (UCSC Xena, SurvivalMeth and MethSurv) to analyze the *Mapk14* methylation levels in CRC patients from different perspectives. From Figure 4, we can conclude that the DNA methylation level of *Mapk14* is reduced in CRC tissues compared with normal colorectal tissues. Notably, *Mapk14* DNA is only locally methylated. To investigate the DNA methylation-specific sites of *Mapk14* and validate the obtained results, we performed an in-depth analysis of the TCGA CRC patient data using the SurvivalMeth and MethSurv tools. A total of three CPG sites located on the CPG island, namely, cg02935305, cg24198611 and cg09065504, indicated poor prognosis. (Figures 5A–C). In addition, we also found that cg05798012 and cg25375420 of *Mapk14* were the most highly methylated sites (Figure 5D). In the COAD-READ tissues, *Mapk14* DNA methylation occurred mainly at the hypomethylation level of the gene body (*p* < 0.01) (Figure 5D; Supplementary Figure S1; Table 1). Therefore, the high protein expression accompanying the hypomethylation of the *Mapk14* gene may affect the oncogenic effect.

**TABLE 1:** Differences in methylation levels of Mapk14 probes between CRC and normal samples.

Probe ID	Gene location	CpG island regions	Average methylated data value of tumor samples	Average methylated data value of normal samples	Delta value	Fold change	*p*-Value
cg24198611	−	Island	0.015854	0.014965	0.000888	1.059383	0.00475688
cg24991452	BODY	Island	0.02897	0.026169	0.002801	1.107036	0.003100358
cg08502873	−	Island	0.029626	0.026444	0.003181	1.120316	0.009894574
cg02935305	TSS1500	Island	0.035297	0.031443	0.003854	1.122571	0.002749216
cg24495832	BODY	S_Shore	0.089389	0.080341	0.009047	1.112614	6.82E-05
cg17498313	TSS200	Island	0.067608	0.058172	0.009435	1.162194	6.98E-06
cg05624487	BODY	S_Shore	0.103563	0.090507	0.013056	1.144254	3.44E-06
cg24991452	BODY	Island	0.027772	0.02247	0.005301	1.235909	0.002216622
cg02412050	TSS200	Island	0.04905	0.040896	0.008154	1.199383	0.00016777
cg17498313	TSS200	Island	0.064692	0.052179	0.012512	1.239803	3.32E-05

### Mapk14 at the Single-Cell Level of Times in CRC

**FIGURE 6:**
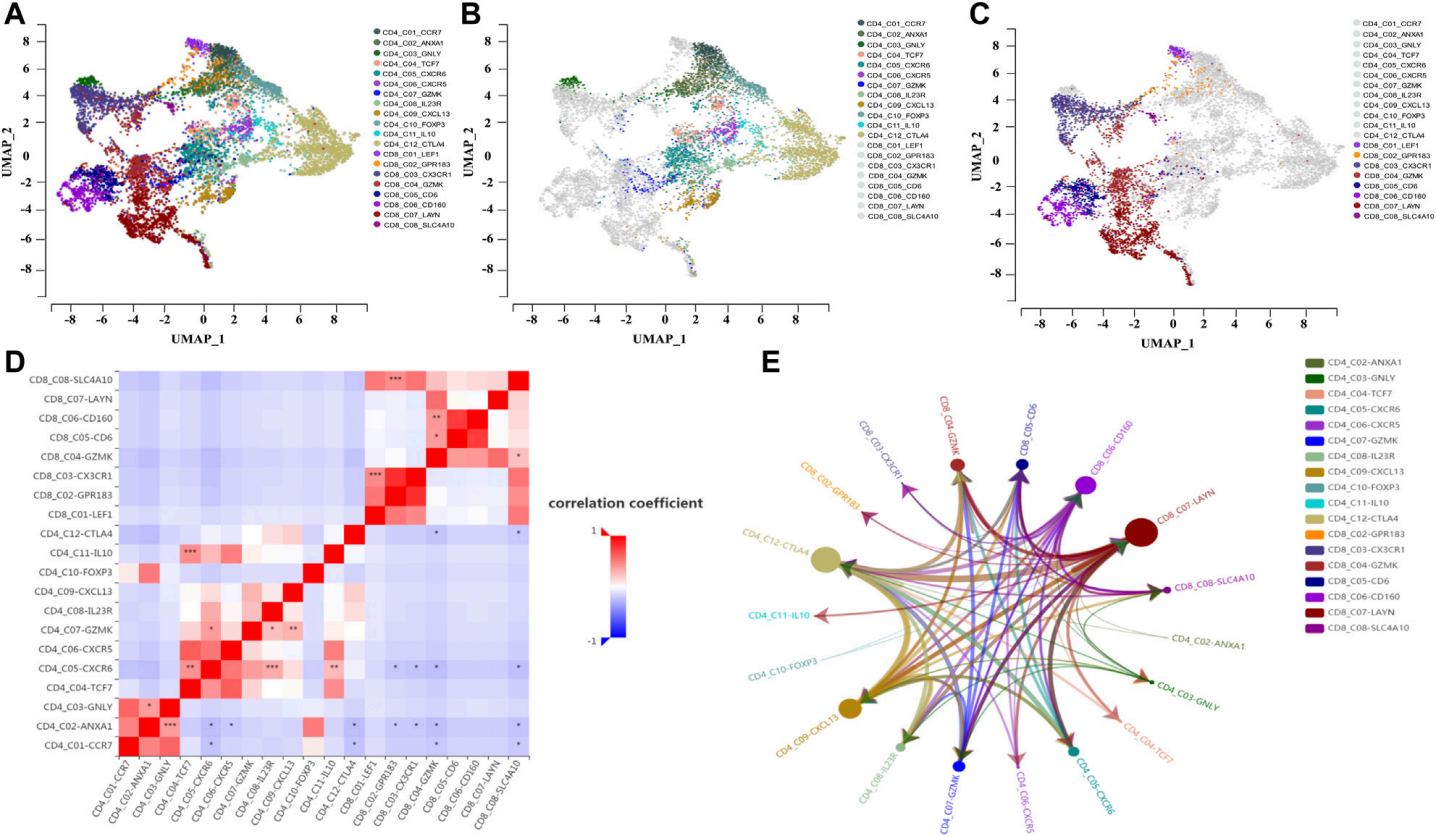
Single-cell analysis of Mapk14 in CRC. **(A)** Clustering of different cell types of CD4/CD8 of Mapk14 in CRC. **(B)** Clustering of different cell types of CD4 of Mapk14 in CRC. **(C)** Clustering of different CD8 cell types of Mapk14 in CRC. **(D)** Correlation of the ratio of different cell types of CD4/CD8 of Mapk14 in CRC. **(E)** Regulatory network between different CD4/CD8 immune cell types of Mapk14 in CRC.

To investigate the interactions between the tumor immune microenvironment and single cells, we used the online scTIME tool for *Mapk14* analysis of tumor immune microenvironment specificity and applied the GSE108989 dataset for single-cell transcriptome visualization in colorectal cancer (Figure 6A). We obtained 12 CD4^+^ T-cell clusters (Figure 6B), namely, *CCR7*, *ANXA1*, *GNLY*, *TCF7*, *CXCR6*, *CXCR5*, *GZMK*, *IL23R*, *CXCL13*, *FOXP3*, *IL10,* and *CTLA4*. CD8^+^ T-cells were divided into 8 clusters (Figure 6C), namely, *LEF1*, *GPR183*, *CX3CR1*, *GZMK*, *CD6*, *CD160*, *LAYN,* and *SLC4A10*. To explore the colorectal cancer specificity of cell types, we calculated the cell numbers of colorectal cancer patients with their immune cell types and the cell proportions (Supplementary Figure S2) and found that *Mapk14* expression significantly differed in CRC patients with different CD4/CD8 cell types (Figure 6D). Among those CD4/CD8 cell types, *Mapk14* expression in *CD4_C03-GNLY* vs. *CD4_C02-ANXA1*, *CD4_C04-TCF7* vs. *CD4_C05-CXCR6* and *CD8_C01-LEF1 vs*. *CD8_C03-CX3CR1* was positively correlated with that in adjacent normal colorectal tissues, peripheral blood and colorectal cancer tumors (Supplementary Figure S3) while that in *CD4_C05-CXCR6* vs. *CD4-C01-CCR7*, *CD4_C06-CXCR5* vs. *CD4-C02-ANXA1* and *CD8_C04-GZMK vs*. *CD4_C01-CCR7* was negatively correlated with that in adjacent normal colorectal tissues, peripheral blood and colorectal cancer tumors (Supplementary Figure S4). These results suggest significant differences in *Mapk14* gene expression in different CD4/CD8 cell types.

Finally, we analyzed the cell-cell communication between different cell types based on the LR-Network fraction from the overall number of significantly interacting ligand-receptors (ligand-receptor interaction strength threshold of 28.284, and *p* value of 0.05). *Mapk14* was highly expressed and ordered as follows:*CD8_C07-LAYN* > *CD4_C12-CTLA4* > *CD4_C09-CXCL13* > *CD8_C06-CD160* (Figure 6E; Supplementary Figure S5).

### Relationship Between Mapk14 Expression and Immune Characteristics in CRC

**FIGURE 7:**
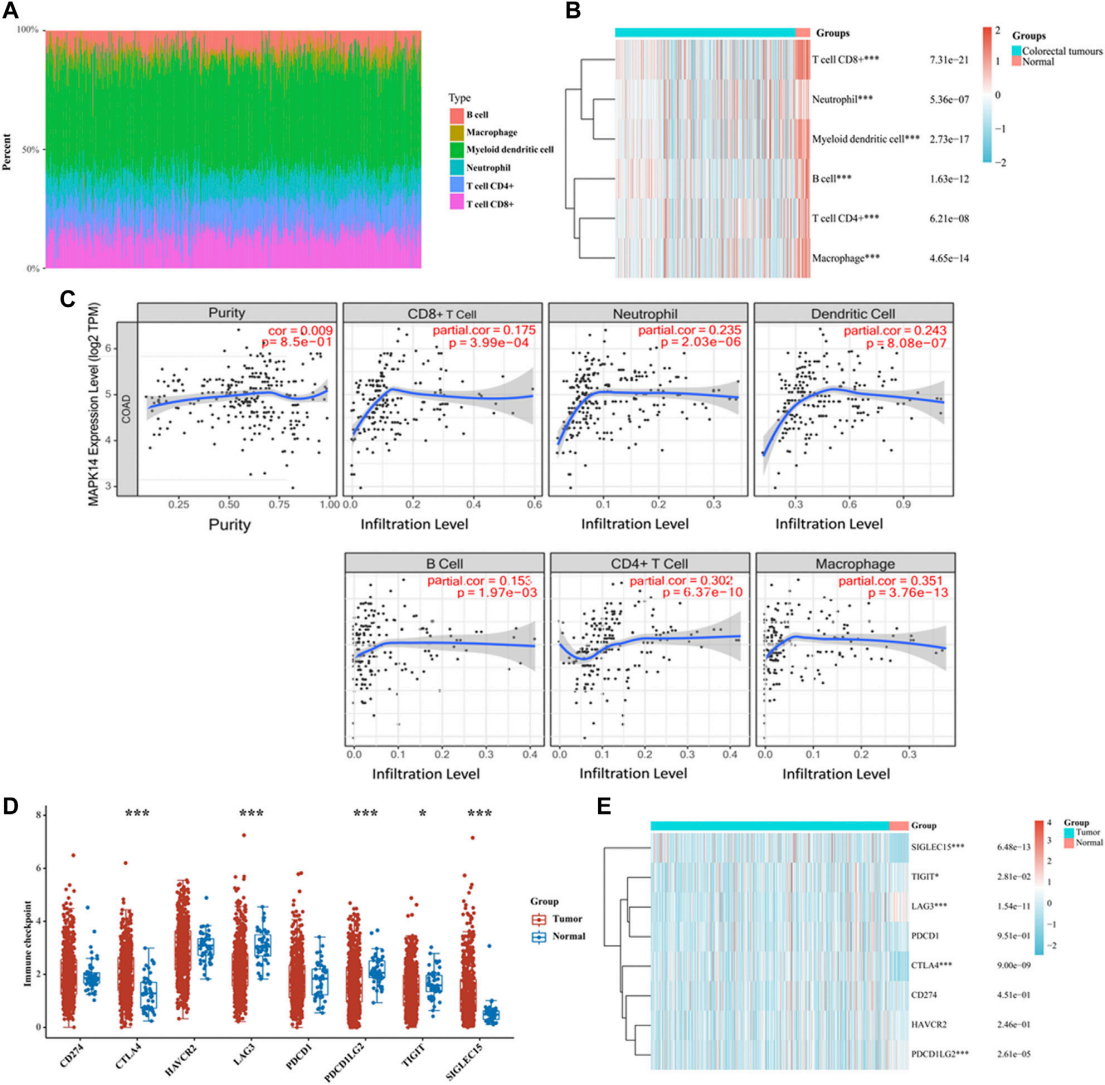
Analysis of Mapk14 expression in CRC, including the immune score and immune checkpoint. **(A)** Percentage abundance of tumor-infiltrating immune cells in CRC samples. Different colors represent different immune cell types. Horizontal coordinates represent samples, and vertical coordinates represent the percentage of immune cell content in individual samples. **(B)** Heat map of immune cell scores. Different colors represent the expression trends in different samples. **(C)** Correlation analysis of Mapk14 expression in CRC with CD8^+^ T cells, neutrophils, dendritic cells, B cells, CD4^+^ T cells and macrophage immune cells. **(D)** Immune checkpoint expression distribution of the Mapk14 gene in CRC and normal tissues. **(E)** Heat map of immune checkpoint-associated gene expression. *p < 0.05, **p < 0.01, ***p < 0.001.

Tumor infiltrating lymphocytes (TILs) are essential components in the tumor microenvironment and exert important regulatory effects on tumors. To better understand how tumor-infiltrating lymphocytes affect CRC tumor *Mapk14* expression and the significance of the effect, a TIMER analysis of TCGA data was performed to assess *Mapk14* gene expression in tumor-infiltrating immune cells in CRC. We first analyzed the percentage abundance of *Mapk14* in CRC with different types of tumor-infiltrating immune cells (Figure 7A). Then, we investigated the correlation between *Mapk14* expression and tumor-infiltrating immune cells by establishing immune cell scoring heat maps, and the results showed that high *Mapk14* expression was significantly associated with immune infiltration in CRC (Figure 7B). Specifically, *Mapk14* expression was not significantly correlated with tumor purity (*p* = 0.85) but significantly positively correlated with the abundance of infiltration of several immune cell types, including CD8^+^ T cells (r = 0.175, *p* = 3.99e-04), neutrophils (r = 0.235, *p* = 2.03e-06), dendritic cells (r = 0.243, *p* = 8.08e-07), B cells (r = 0.153, *p* = 1.97e-03), CD4+T cells (r = 0.302, *p* = 6.36e-10) and macrophages (r = 0.351, *p* = 3.76e-13) (Figure 7C). Taken together, these results showed that *Mapk14* expression was significantly correlated with tumor immune infiltration.

Targeting tumor immune checkpoints to modulate tumors is considered a promising novel tumor treatment modality that has ushered in a new era of tumor immunotherapy. Subsequently, we analyzed the expression of *Mapk14* and *SIGLEC15*, *TIGIT*, *CD274*, *HAVCR2*, *PDCD1*, *CTLA4*, *LAG3* and *PDCD1LG2* immune checkpoint-related genes. The results revealed that *Mapk14* expression was significantly associated with the *SIGLEC15*, *TIGIT*, *LAG3*, *CTLA4* and *PDCD1LG2* immune checkpoint markers (Figures 7D,E). Notably, *CTLA4* is a biomarker of the response to immune checkpoint inhibitors (Zhu et al., 2018) and is significantly correlated with *Mapk14* expression in CRC. These analyses provide strong evidence that the *Mapk14* gene is associated with immune characteristics.

### Drug Sensitivity Analysis of TMB, MSI and Mapk14

**FIGURE 8:**
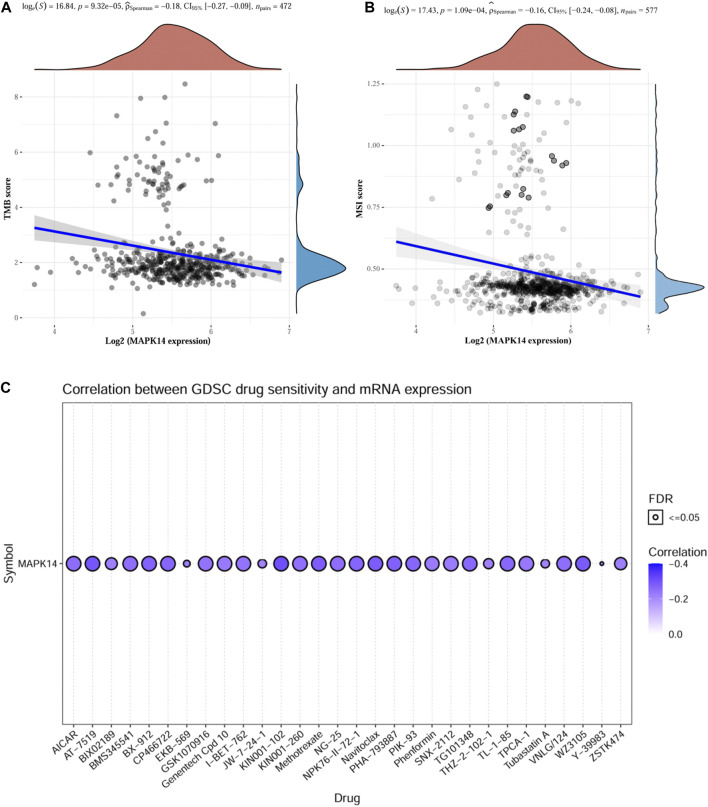
TMB, MSI and drug sensitivity analyses of Mapk14 in CRC. **(A)** Correlation between Mapk14 and TMB in CRC. **(B)** Correlation between CRC and MSI. **(C)** Correlation between Mapk14 and CTRP drug sensitivity.

TMB can be used as a biomarker to predict the efficacy of immunotherapy in colorectal cancer (Vanderwalde et al., 2018), while MSI is considered a predictive biomarker for cancer immunotherapy (Chang et al., 2018). The above findings suggest that *Mapk14* is significantly associated with tumor immune infiltration. To elucidate whether *Mapk14* could also be used as a biomarker for drug screening, we analyzed the correlation between *Mapk14* expression and TMB and MSI. The results showed a negative correlation between *Mapk14* and TMB [*p* = 9.32 e-05, 95% CI (−0.27, −0.09)] (Figure 8A) and between *Mapk14* expression and MSI [*p* = 1.09 e-04, 95% CI (−0.24, −0.08)] (Figure 8B). To investigate whether *Mapk14* can be used as a therapeutic target for development, it is important to analyze the correlation between *Mapk14* gene expression and drug responses. A drug sensitivity analysis was performed, and it showed that the expression of *Mapk14* was positively correlated with most drugs (Figure 8C), which further confirmed that drugs targeting *Mapk14* may play a therapeutic role in colorectal cancer.

### Immunohistochemical (IHC) Validation of Mapk14 Expression in Clinical Specimens of Colorectal Cancer and Normal Colorectal Tissues

**FIGURE 9:**
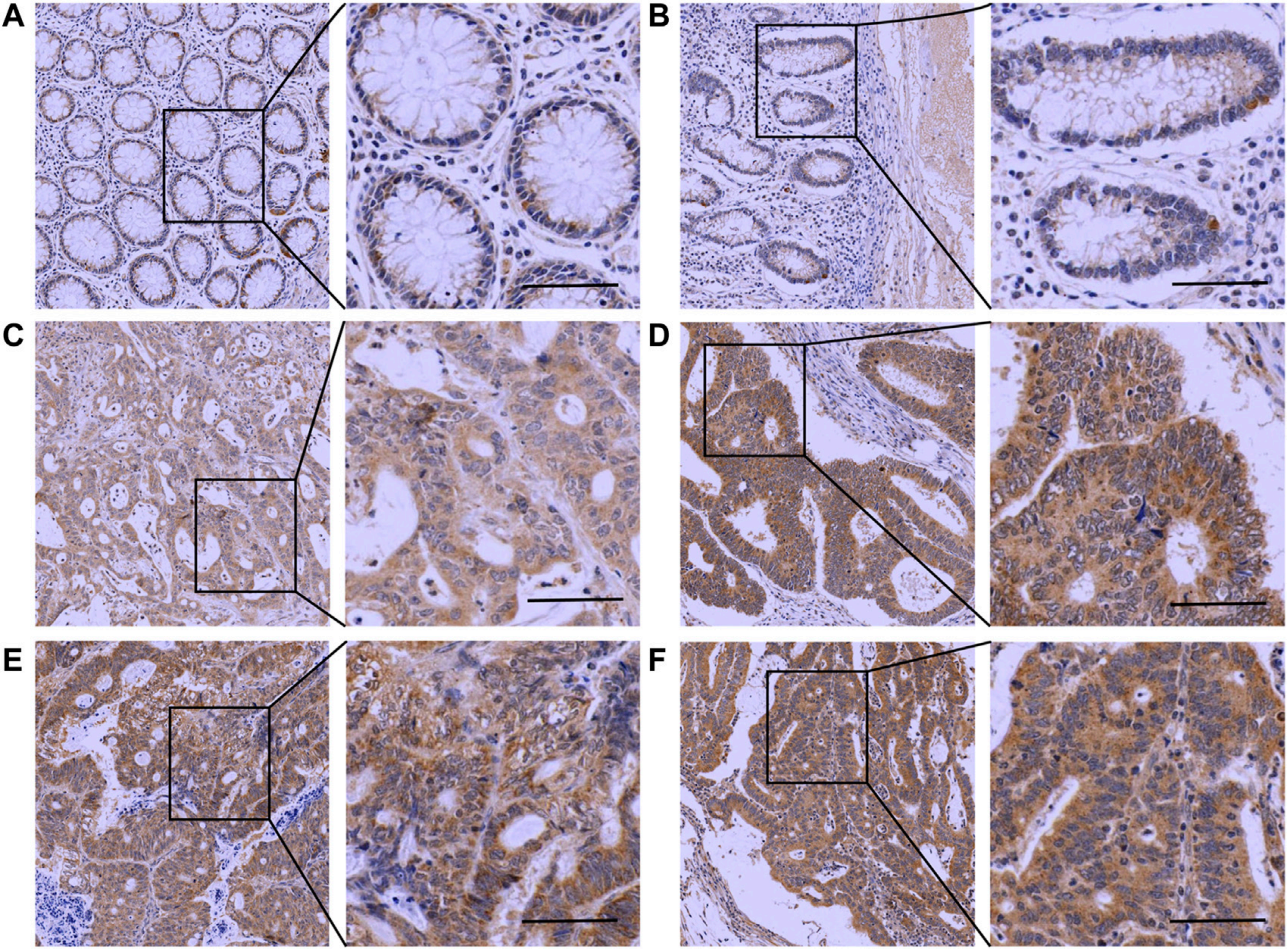
Immunohistochemical analysis of Mapk14 expression in CRC tissues. **(A, B)** Mapk14 expression in normal tissues. **(C)** Mapk14 expression in CRC tumor tissues at stage I. **(D)** Mapk14 expression in CRC tumor tissues at stage II. **(E)** Expression of Mapk14 in CRC tumor tissues at stage III. **(F)** Expression of Mapk14 in CRC tumor tissues at stage IV. (Left: SP × 200; Right: magnified view).

**FIGURE 10:**
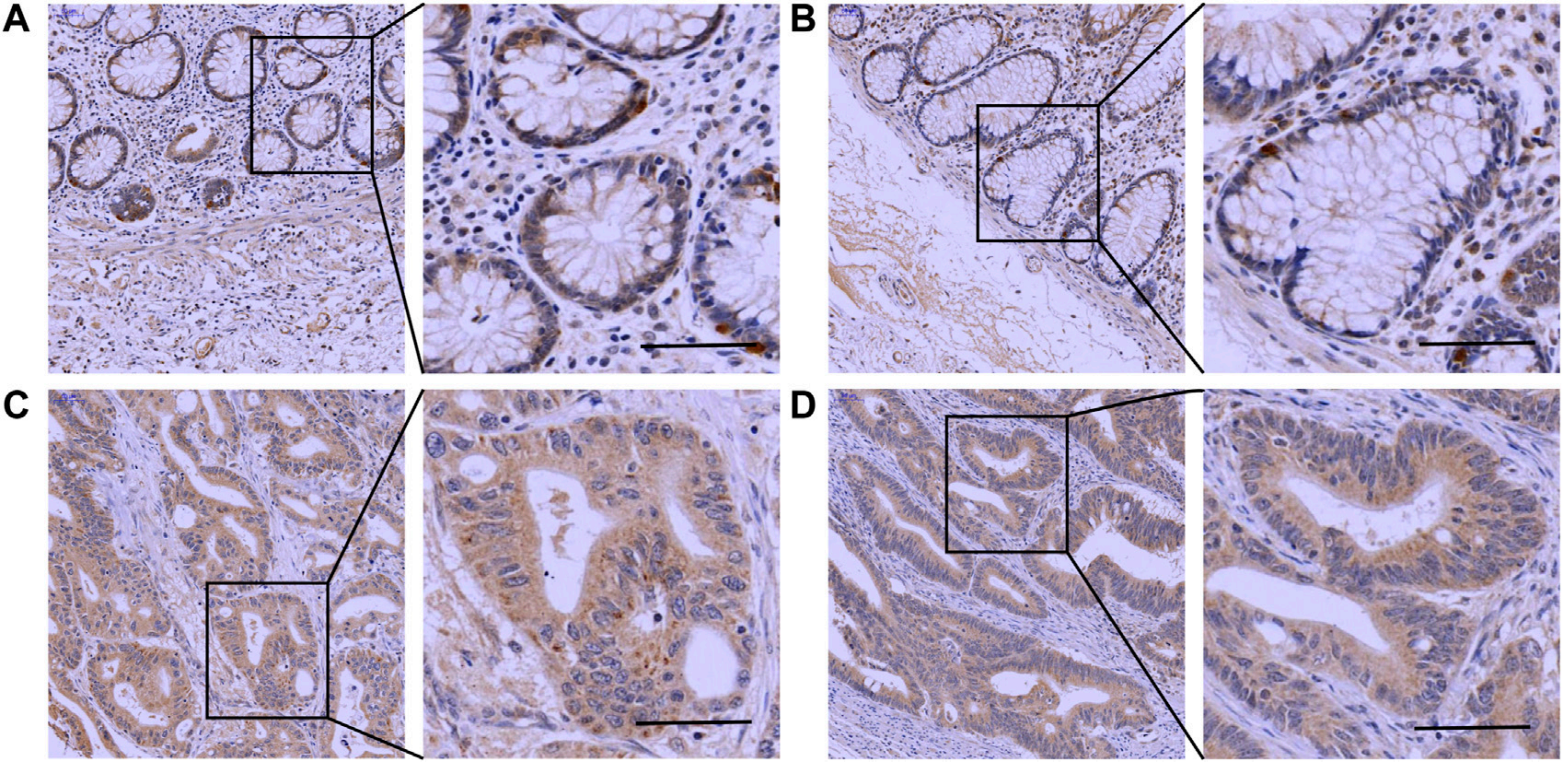
Immunohistochemical validation of Mapk14 expression in M-stage colorectal cancer tissues and normal tissues. **(A,B)** Mapk14 expression in normal tissues. **(C)** Mapk14 expression in M0-stage CRC tumor tissues. **(D)** Expression of Mapk14 in M1-stage CRC tumor tissues. (Left: SP×200; Right: magnified view).

The IHC results showed that *Mapk14* was expressed at higher levels in the colorectal cancer tissue of patients with clinical stages I, II, III and IV than in normal tissues (Figure 9; Table 2). In addition, the *Mapk14* protein expression levels were significantly higher in colorectal cancer tissues at the M0 and M1 stage than in normal tissues adjacent to cancer in the M stage (Figure 10). These results were generally consistent with the bioinformatics analysis results and indicated that high expression of *Mapk14* contributed to the occurrence and development of colorectal cancer*.*

**TABLE 2:** Immunohistochemical detection of Mapk14 protein expression in CRC and paraneoplastic tissues.

Sample	P38α(Mapk14)		*χ* ^*2*^	*P*
	Positive	Negative		
Cancer tissue	31 (88.6%)	4 (11.4%)	16.232	0.000
Paracancerous tissue	15 (42.9%)	20 (57.1%)		

## Discussion

Colorectal cancer is a common malignancy of the gastrointestinal tract, and although the main treatment options include combined surgery, chemotherapy, and radiotherapy, the prognosis of patients with colorectal cancer is still poor. In recent years, cancer immunotherapy has changed the paradigm of cancer treatment and is gradually being considered a promising strategy for the treatment of certain cancers (Riley et al., 2019). Existing tumor immunotherapies include cancer vaccines, cellular immunotherapy, and immune checkpoint inhibitors, and several clinical trials of related therapies have been conducted for CRC. However, these treatments only have a significant effect for certain patients. Moreover, the response efficiency of most patients is low. Therefore, new immune infiltration-related biomarkers are urgently needed to predict the prognosis of patients and identify the underlying molecular mechanisms of the immunotherapeutic response.

Growing evidence has shown that *Mapk14* plays a critical role in the occurrence and progression of multiple human cancers, including CRC. However, studies of the relationship between the *Mapk14* gene and CRC are limited; thus, we decided to perform a comprehensive and integrated bioinformatics analysis. To the best of our knowledge, few studies have explored *Mapk14* expression and its potential prognostic and tumor microenvironmental impact on CRC; therefore, the potential role of *Mapk14* in CRC is the focus of this study.

We first elucidated the expression and prognostic value of *Mapk14* in CRC and found that *Mapk14* mRNA expression was higher in multiple cancers than in normal tissues, including COAD-READ tissues. In CRC, *Mapk14* is highly expressed in a variety of tumor cell lines. The results of the clinicopathological feature assessment showed that *Mapk14* expression was significantly associated with the clinical stage (I, II, III and IV) and M stage. Additionally, abnormally high *Mapk14* expression was associated with poorer clinical characteristics in the CRC cohort. In addition, our results showed that CRC patients with high *Mapk14* expression exhibited greater recurrence and poorer prognoses; therefore, *Mapk14* can be used as an oncogene in CRC. Numerous studies have confirmed that abnormal DNA methylation occurs throughout the tumor occurrence and development process and detected abnormal methylation changes in the early tumor formation stage and during disease progression and late tumor recurrence and metastasis (Werner et al., 2017). In the study of nasopharyngeal carcinoma, it is found that the RAS association domain family 1A (RASSF1A) gene and disheveled-associated binding antagonist of β-catenin 2 (*DACT2*) promoter methylation can be used as markers for early auxiliary diagnosis (Ye et al., 2017; Zhang et al., 2018). In the Colorectal Cancer Study, a meta-analysis involving 24 articles and 2025 patients reported that *APC* gene promoter hypermethylation is an early event in colorectal carcinogenesis and can be used as a diagnostic indicator for early colorectal cancer (Liang et al., 2017). We further analyzed the methylation of *Mapk14*, and the results showed that *Mapk14* was hypomethylated in the genome. The main factors affecting *Mapk14* mRNA expression in colorectal cancer are DNA methylation and CNV alteration, while gene mutation is not a major factor. This change occurs gradually in CpG islands and is age-related, and it can also be observed in the early stage of tumorigenesis. In conclusion, *Mapk14* is highly expressed, and its genome is hypomethylated in CRC, which may be the main mechanism underlying its gene expression.

Stromal cells in the tumor microenvironment can alter the oncogenic properties of tumor cells. Among them, T cells, B cells, natural killer cells and other types of lymphocytes also play an important role in the tumor immune microenvironment (Albini and Sporn, 2007). However, single-cell RNA sequencing can provide favorable conditions for depicting the state of immune cells in the tumor microenvironment at the single-cell level, and it is easier to observe and identify new subsets of tumor-related immune cells (Golsaz-Shirazi et al., 2018). Several factors that affect the treatment response of immune checkpoints have been identified, and they include tumor antigens (such as tumor mutation load and microsatellite instability) (Wang et al., 2017) and immunosuppressive and inflammatory cells or proteins (such as TILs and tumor-related immune cells, gene markers, *CTLA-4*, *IDO* and *PD-L1*) (Cristescu et al., 2018). These indicators can be used to predict the effectiveness of immunotherapy. Our results showed that *Mapk14* expression is significantly correlated with different cell types and tumor immune cell infiltration according to the scTIME and TIMER databases. In addition, *Mapk14* expression was significantly correlated with the immune score and immune checkpoint in colorectal cancer. It is worth highlighting that *Mapk14* expression was negatively correlated with TMB and MSI but positively correlated with most therapeutic agents, thus reinforcing the possibility that *Mapk14* may enable individualized treatment for optimal clinical benefit.

Further immunohistochemical analysis of *Mapk14* expression at different clinical stages and M stages showed that *Mapk14* was significantly highly expressed in tumor tissues, which further suggests the potential use of *Mapk14* as a marker of CRC progression. In conclusion, our findings revealed that *Mapk14* has a predictable effect on clinical features and is associated with the immune microenvironment of colorectal cancer. Thus, *Mapk14* could serve as a new prognostic biomarker and potentially advance the development of new immunotherapeutic strategies.

Althought our research revealed that *Mapk14* can be used as a colorectal cancer biomarker, there are still further experiments and analysis that need to be considered. For example, we did not determine the optimal cut-off value of *Mapk14*. Here, the median Mapk14 mRNA expression was considered as the cut-off value. Furthermore, the relationship between *SIGLEC15,TIGIT,LAG3,CTLA4, PDCD1LG2* immune checkpoints and *Mapk14* was only verified by correlation analysis, and we need to further explore the mechanism of *Mapk14* expression in colorectal cancer *in vitro* and vivo experiments, such as single cell RNA sequencing.

## Conclusion

In conclusion, this study confirms that *Mapk14* is an oncogene that is highly expressed in colorectal cancer clinicopathology. This study demonstrates that *Mapk14* is a prognostic biomarker for the clinicopathological features and tumor immune microenvironment of CRC and has potential as a predictive biomarker and immunotherapy target.

## Data Availability

The original contributions presented in the study are included in the article/Supplementary Material, further inquiries can be directed to the corresponding authors.

## Footnotes

1
https://portal.gdc.cancer.gov/

2
https://portals.broadinstitute.org/ccle

3
https://commonfund.nih.gov/GTex

4
https://www.ncbi.nlm.nih.gov/geo/

5
http://xena.ucsc.edu/

6
http://biobigdata.hrbmu.edu.cn/survivalmeth/

7
https://biit.cs.ut.ee/methsurv/

8
http://scTIME.sklehabc.com

9
https://cistrome.shinyapps.io/timer/
