# Supplementary figures and images for: Mapk14 is a Prognostic Biomarker and Correlates with the Clinicopathological Features and Immune Infiltration of Colorectal Cancer
Source: Front Cell Dev Biol. 2022 Jan 24;10:817800. doi: 10.3389/fcell.2022.817800 (PMC8818961; doi:10.3389/fcell.2022.817800)

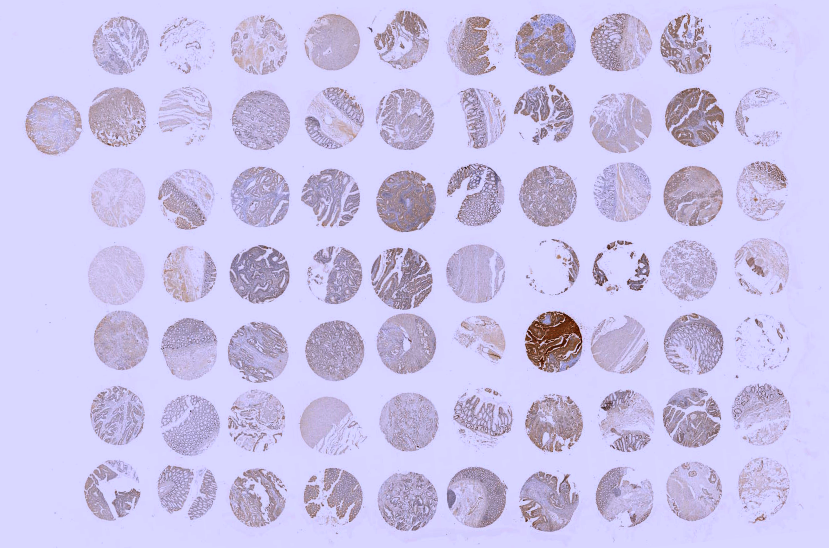

Supplement: Supplementary file 3 [file Image6.TIF]

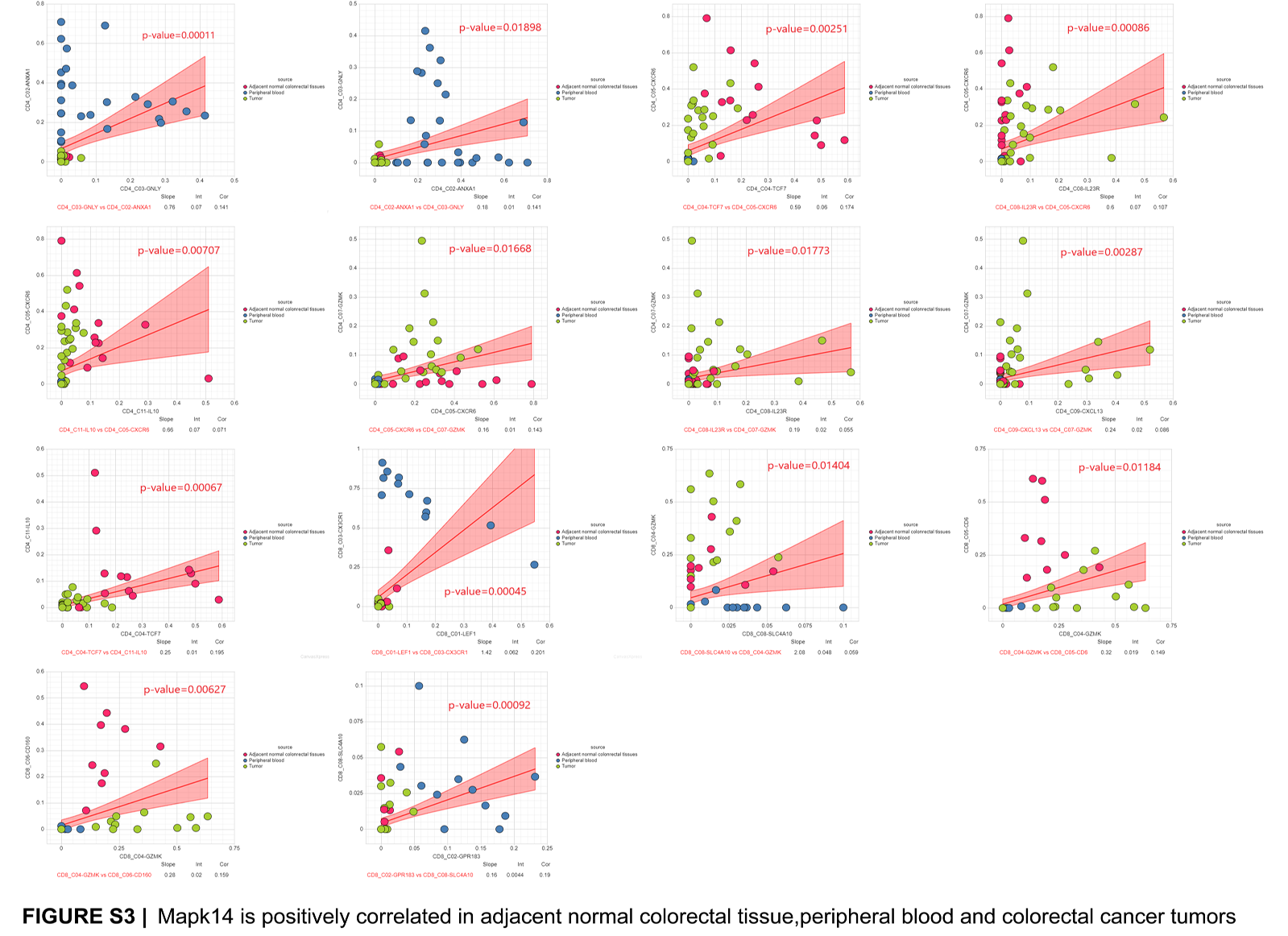

Supplement: Supplementary file 5 [file Image3.TIF]

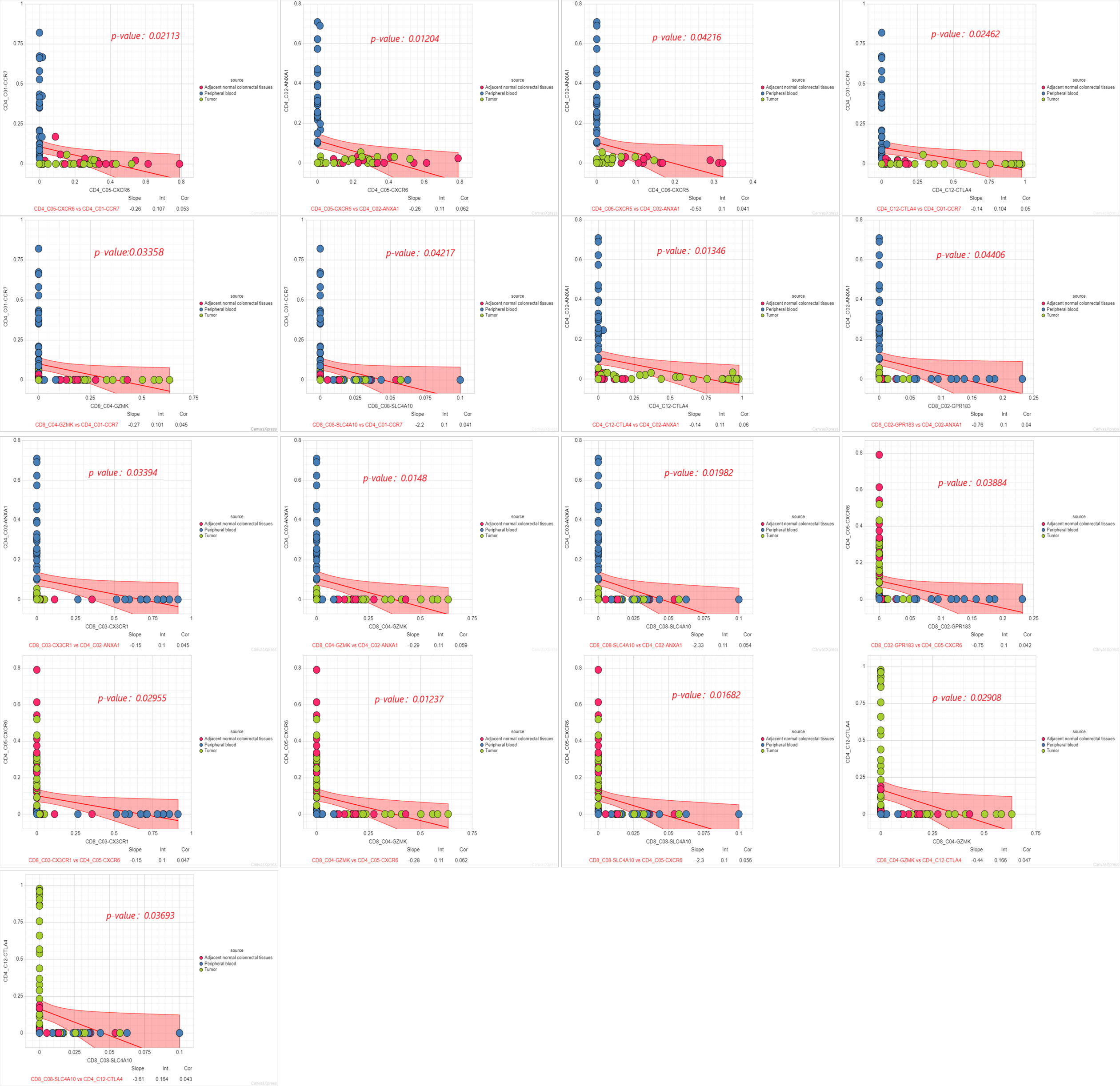

Supplement: Supplementary file 6 [file Image4.TIF]

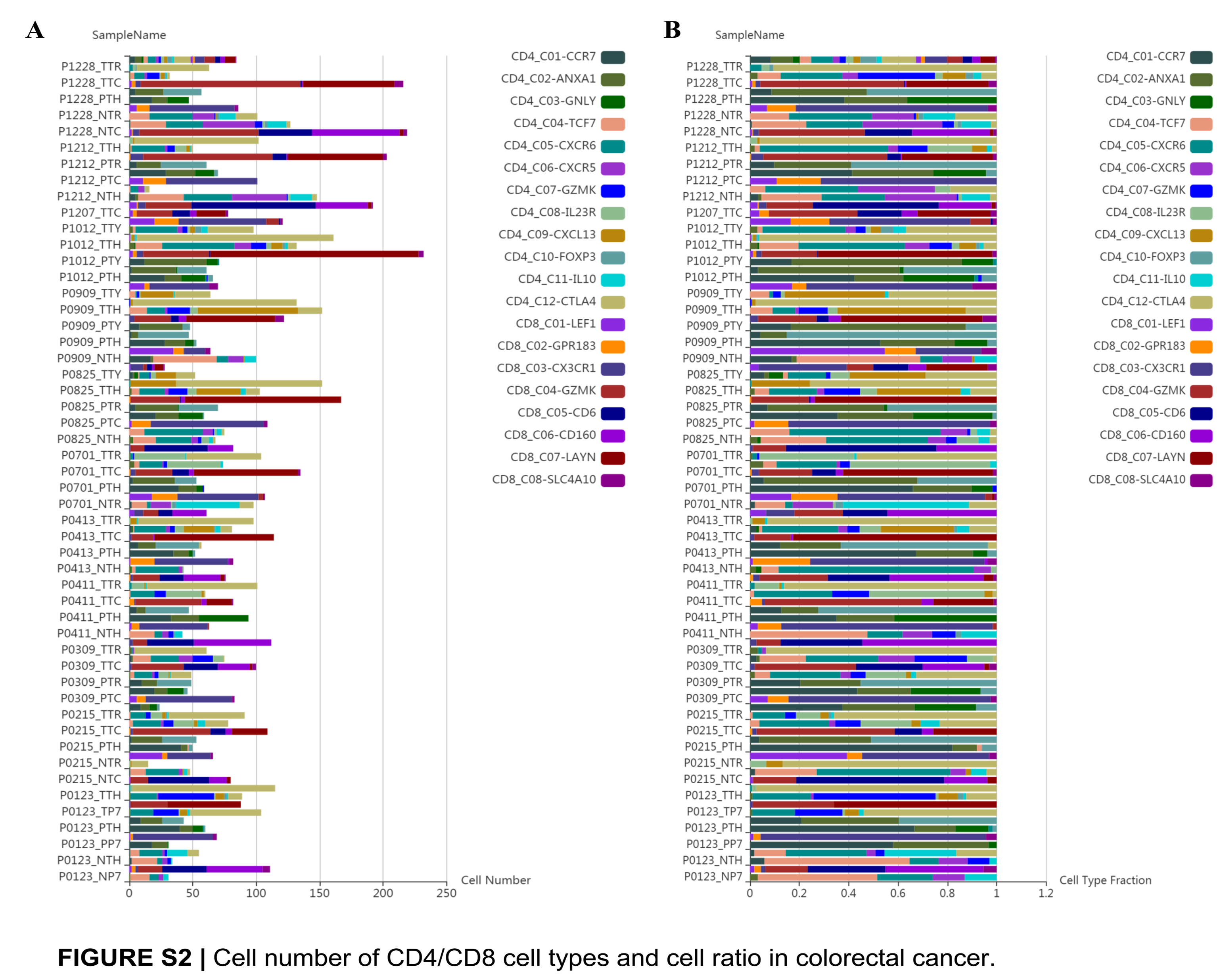

Supplement: Supplementary file 7 [file Image2.TIF]

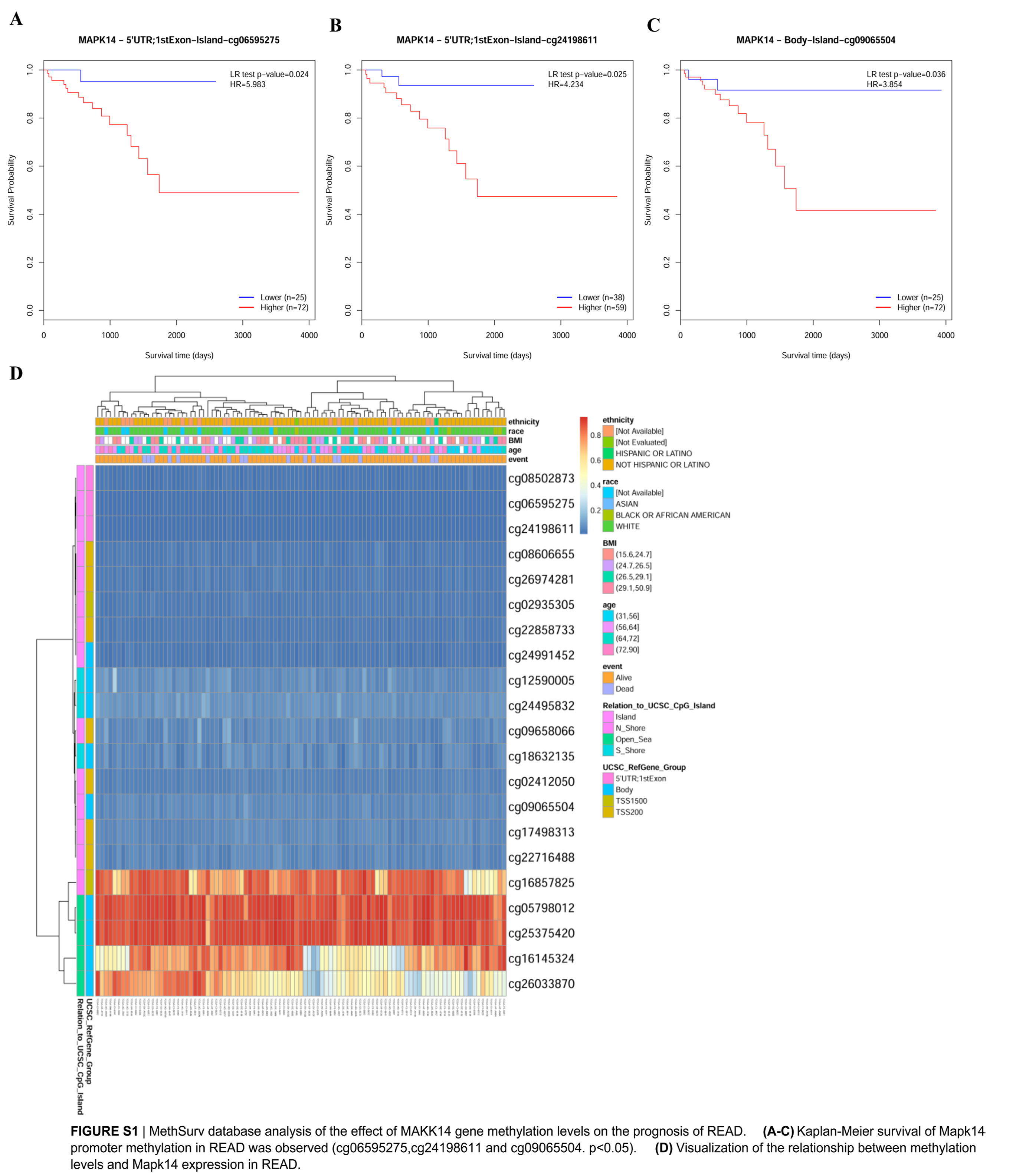

Supplement: Supplementary file 8 [file Image1.TIF]

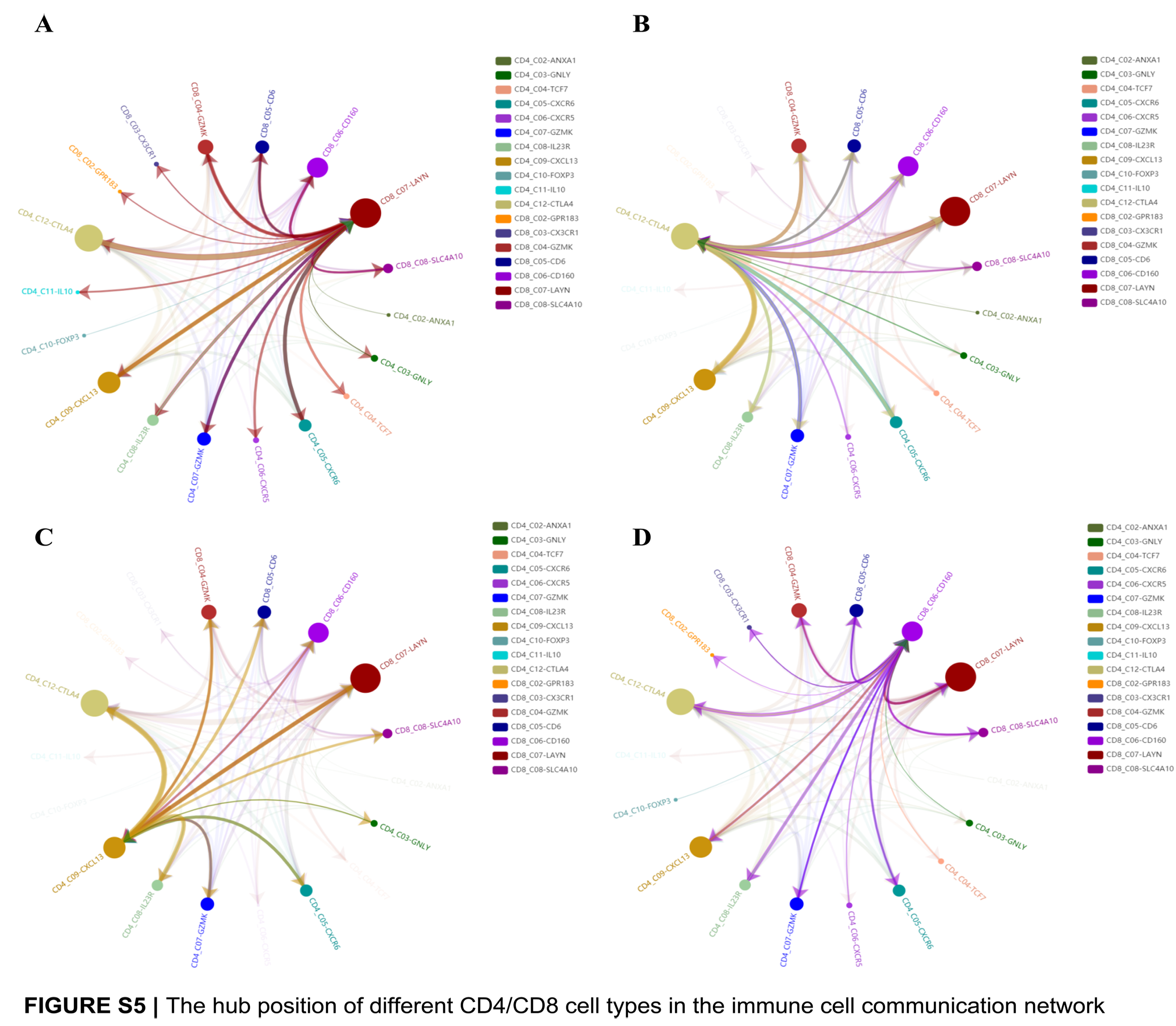

Supplement: Supplementary file 12 [file Image5.TIF]
